# Earthworms increase forest litter mass loss irrespective of deposited compounds – A field manipulation experiment in subtropical forests

**DOI:** 10.1002/ece3.10047

**Published:** 2023-04-30

**Authors:** Junbo Yang, Kai Tian, Jingzhong Lu, Xiangshi Kong, Qiang Li, Rumeng Ye, Xiaoyi Zeng, Tingting Cao, Haijing Hu, Yanli Ji, Xingjun Tian, Stefan Scheu

**Affiliations:** ^1^ State Key Laboratory of Pharmaceutical Biotechnology, School of Life Sciences Nanjing University Nanjing China; ^2^ Johann‐Friedrich‐Blumenbach Institute of Zoology and Anthropology University of Göttingen Göttingen Germany; ^3^ College of Life Science and Agricultural Engineering Nanyang Normal University Nanyang China; ^4^ Key Laboratory for Ecotourism of Hunan Province, School of Tourism and Management Engineering Jishou University Jishou China; ^5^ College of Eco‐Environmental Engineering Qinghai University Xining China; ^6^ Center of Biodiversity and Sustainable Land Use University of Göttingen Göttingen Germany

**Keywords:** aboveground–belowground linkages, atmospheric depositions, Brown food web, carbon cycling, *Eisenia fetida*, subtropic forests

## Abstract

Earthworms modulate carbon and nitrogen cycling in terrestrial ecosystems, but their effect may be compromised by the deposition of pollutants from industrial emissions. However, studies investigating how deposited compounds affect the role of earthworms in carbon cycling such as litter decomposition are lacking, although the interactions of earthworms and deposited compounds are important for understanding the impact of pollutants on ecosystems and the potential of earthworms in bioremediation. We performed a 365‐day in situ litterbag decomposition experiment in a deciduous (*Quercus variabilis*) and coniferous (*Pinus massoniana*) forest in southeast China. We manipulated nitrogen (N), sodium (Na), and polycyclic aromatic hydrocarbons (PAHs) as model compounds during litter decomposition with and without earthworms (*Eisenia fetida*). After one year, N, Na, and PAH all slowed down litter mass loss, with the effects of Na being the strongest. By contrast, *E. fetida* generally increased litter mass loss, and the positive effects were uniformly maintained irrespective of the type of compounds added. However, the pathways to how earthworms increased litter mass loss varied among the compounds added and the two forests studied. As indicated by structural equation modeling, earthworms mitigated the negative effects of deposited compounds by directly increasing litter mass loss and indirectly increasing soil pH and microbial biomass. Overall, the results indicate that the acceleration of litter mass loss by earthworms is little affected by deposited compounds, and that earthworms have the potential to mitigate negative impacts of pollutants on litter decomposition and ecosystem processes.

## Graphical abstract

The addition of N, Na, and PAHs decreased litter mass loss. Earthworms uniformly increased forest litter mass loss irrespective of the type of deposited compounds added. The pathways to how earthworms promoted litter mass loss varied with N, Na, and PAH treatments.

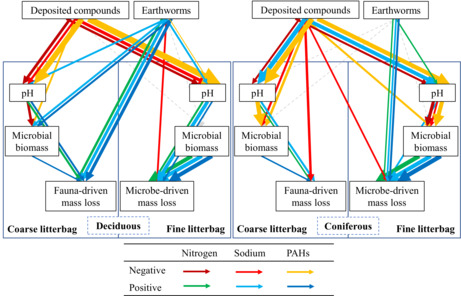

## 1 INTRODUCTION

Human activities such as industrial emissions increase the input of pollutants into natural ecosystems, thereby affecting major processes of carbon cycling such as litter decomposition (Knorr et al., 2005). In terrestrial ecosystems, litter decomposition is accelerated by soil decomposers such as earthworms (Cortez, 1998). Although the effects of deposited compounds and earthworms on litter decomposition were studied separately, their interactive effect remains elusive (Huang et al., 2020; Zhang et al., 2018). This gap limits our understanding of the influence of deposited compounds on terrestrial ecosystems and the role of earthworms under the increasing input of deposited compounds. Filling this gap is also important for bioremediation, e.g., by using earthworms for the remediation of contaminated soils.

Earthworms as major soil detritivores form part of the soil macrofauna. They contribute to litter decomposition by directly fragmenting litter and indirectly influencing soil properties, and the structure and activities of microorganisms and other fauna (Kizilkaya et al., 2011; Sackett et al., 2013; Schulmann & Tiunov, 1999). *Eisenia fetida* was found abundant in compost heaps where it accelerates organic matter decomposition by stimulating microbial activities and affecting soil pH (Aira et al., 2006; Das et al., 2022). However, *E. fetida* also lives in forests in southern Europe and eastern China, in particular close to urban areas (Huang et al., 2003). This epigeic species predominantly colonizes the litter layer and is known for its resistance to soil contaminants (Rodriguez‐Campos et al., 2014). Although well known for its acceleration of litter decomposition and composting processes, modifications of the effects of *E. fetida* on litter decomposition by deposited compounds received little attention. This is unfortunate as it hampers the use of *E. fetida* and other epigeic earthworms for the bioremediation of contaminated soils.

Atmospheric depositions are changing terrestrial ecosystems at global scales (Holland et al., 2005; Liu et al., 2011). Industrial emissions increase the input of a variety of compounds into forest ecosystems including e.g., nitrogen (N), sodium (Na), and polycyclic aromatic hydrocarbons (PAHs; Li et al., 2016; Wang et al., 2015). In urban regions, road salt also increases the Na content in soil (Tiwari & Rachlin, 2018). Although typically deposited at low rates, each N, Na, and PAHs have been shown to affect litter mass loss by influencing soil microbial and faunal activity (Kaspari et al., 2014; Knorr et al., 2005; Qasemian et al., 2012), with their effects increasing at higher concentrations (Gruntz et al., 2022; Zhang, Chao, et al., 2016; Zhang, Mora, et al., 2016).

The effects of deposited compounds on litter decomposition may be due to a number of pathways. For example, N addition may promote soil acidification and thereby detrimentally affecting soil microbial activity but little affecting the activity of soil fauna (Lin et al., 2017; Sinsabaugh et al., 2002; Zhang, Chao, et al., 2016); Na addition may meet the Na shortage of soil fauna and increase microbial activity in inland forests (Jia et al., 2015; Kaspari et al., 2014); PAHs affect soil microbial and faunal decomposers by altering habitat and direct toxicity (Blakely et al., 2002; Cotrufo et al., 2014; Klamerus‐Iwan et al., 2015). Although the effects of N, Na, and PAHs on litter mass loss received considerable attention (Kaspari et al., 2014; Knorr et al., 2005; Qasemian et al., 2012), modifications of their effects by earthworms have not been investigated. This, however, is important for understanding the interactive effects of deposited compounds and soil keystone species on nutrient cycling in terrestrial ecosystems.

Here, we explored how the effects of earthworms on litter mass loss interact with high amounts of deposited compounds including N, Na, and PAHs. We performed an in‐situ litter decomposition experiment in deciduous (*Quercus variabilis*) and coniferous (*Pinus massoniana*) forests with and without the addition of the earthworm species *E. fetida* in Eastern China. During 1 year, we measured litter mass loss, total carbon (C) and N loss, soil pH, and soil microbial biomass. To better understand the pathways of how earthworms alter the effects of deposited compounds on litter mass loss, we applied structural equation modeling. We hypothesized that (1) each the addition of N, Na, and PAHs decrease litter mass loss; (2) the positive effect of earthworms on litter mass loss is compromised by the addition of N, Na, and PAHs, and (3) soil pH and microbial biomass function as drivers of litter mass loss, but their role is reduced by N, Na, and PAHs addition, whereas it is enhanced by earthworms.

## 2 MATERIALS AND METHODS

### 2.1 Study sites

The experiment was conducted at a subtropical forest in Zijin Mountain between April 2018 and May 2019 (32°4′ N, 118°51′ E; Nanjing, Eastern China). The forest is mainly covered by *Quercus variabilis* and *Pinus massoniana* (Lin et al., 2017), and the herb layer is dominated by *Parthenocissus quinquefolia* or graminoids, mainly *Carex* spp. (Tian et al., 2018). The mean annual rainfall and air temperature of the subtropical monsoon climate are 1106 mm and 15.4°C, respectively. The rainfall is concentrated in summer (on average 163 mm per month between June and August) and low in winter (on average 50 mm per month between October and March). The bedrock is sandstone and shale, with a humus layer rich in organic matter and nutrients. For a more comprehensive understanding of the role of deposited compounds and earthworms in different forests, we selected a deciduous *Q. variabilis* forest and a coniferous *P. massoniana* forest. These forests, however, were not replicated and analyzed as separate experiments (see below). The two forests were about 900 m away from each other and located at similar altitudes (65 and 175 m, respectively). More details on site conditions are given in Table 1.

**TABLE 1:** Site conditions and litter traits of the deciduous and coniferous forests studied; means ± SD.

Site conditions	Deciduous	Coniferous
Latitude (°N)	32.0555	32.0635
Longitude (°E)	118.8736	118.8727
Elevation (m a.s.l.)	65	175
Slope (°)	5	15
Soil pH	5.30 ± 0.19a	5.62 ± 0.48a
Soil moisture (%)	24.25 ± 1.86a	21.48 ± 0.40b
Soil C (g kg^−1^)	20.30a	15.40b
Soil N (g kg^−1^)	1.30a	1.10a
Soil C: N	15.52a	14.39a
**Litter traits**	*Quercus variabilis*	*Pinus massoniana*
Lignin (%)	31.20 ± 1.08a	40.60 ± 0.77b
Total C (%)	49.50 ± 0.83a	51.30 ± 1.26b
Total N (%)	1.27 ± 0.07a	0.85 ± 0.08b
Lignin:N	33.31 ± 1.77a	60.53 ± 5.77b
C:N	24.63 ± 2.68a	48.47 ± 6.86b

*Note*: Letters indicate significant differences; *t*‐test (*p* < .05, *n* = 5). Values of soil C, soil N, and soil C/N ratio were taken from Tian et al. (2018).

### 2.2 Experimental setup

In the deciduous and coniferous forest, we identified an area of 30 × 20 m for establishing the experiment. Two factors, the type of deposited compounds (control, N, Na, and PAHs) and earthworms (with and without) were investigated. In each forest, each factor (deposited compounds and earthworms) was replicated four times resulting in 32 experimental units comprising individual mesocosms, which were installed from February to April 2018 (for details see Figure S1). For installation, we first dug up an area of 0.5 × 0.5 m to a depth of 0.2 m, i.e., a volume of about 50 L. From the excavated litter and soil material we hand‐sorted earthworms and picked herb seedlings. Then, we placed a 1 × 1 m nylon bag into the pit (0.16 mm mesh size) and filled back the excavated soil (without or with reduced numbers of earthworms and seedlings). The nylon bags were then closed by a zipper at the top and covered with soil and litter to fit the natural layering of the forest floor (Figure S1c). The pits were located away from the main roots of trees. Individual mesocosms were established as fast as possible (<30 min) to minimize the effects of sunshine and drying. The nylon bags prevented colonization by macrofauna including earthworms. The distance between mesocosms was 3–5 m, and mesocosms were located at least 20 m away from the border of the forest to avoid edge effects.

After finalizing the establishment of the mesocosms, we placed 10 litterbags (20 × 10 cm) on the soil surface within each mesocosm (Figure S1d). Litterbags were either filled with intact litter of *Q. variabilis* or *P. massoniana*, which were collected from December 2017 to January 2018 and dried at 40°C for 1 month (for litter traits see Table 1). To disentangle the effects of microorganisms and soil fauna, two mesh sizes were used, a fine mesh of 0.2 mm and a coarse mesh of 5 mm (Yin et al., 2022). The fine and coarse mesh litterbags were filled with 4 and 8 g litter (dry mass), respectively. The higher amount in the large mesh size litterbags was used as we assumed the litter to be decomposed faster due to access by macrofauna. The litterbags were exposed for 365 days from April 2018 to April 2019.

To manipulate deposited compounds, we added 500 mL of aqueous solutions of NH_4_NO_3_, NaCl, and PAH to the N, Na, and PAH treatments every 35 days. Control mesocosms received 500 mL of distilled water. The aqueous solutions of NH_4_NO_3_ were added following Lin et al. (2017) and were equivalent to the mean annual deposited amount of N in the region of Nanjing (47 kg N ha^−1^ y^−1^). NaCl was added at a Na mass percentage of 0.5% and was equivalent to a rate of 39.36 g Na m^−2^ y^−1^. The Na addition followed Jia et al. (2015) simulating Na input due to road salt (Li et al., 2016; Tiwari & Rachlin, 2018). For PAHs, we included fluoranthene (Flu), pyrene (Pyr), chrysene (Chr), benzo[a]pyrene (BaP), and phenanthrene (Phe). These PAHs account for 54% of the mass of 16 prioritized PAHs in the soil of the urban region of Nanjing and Zijin Mountain (Wang et al., 2015). We added a total of 128 mg PAHs per microcosm per year, which is equivalent to 1.813 μg g^−1^ dry soil y^−1^, thereby doubling the total amount of soil PAHs at our study sites (Wang et al., 2015).

For investigating the actual effect of earthworms in the field, treatments with earthworms received a total of 60 individuals of *E. fetida* per mesocosm in the deciduous and 20 individuals in the coniferous forest resembling the density in these forests as investigated in 2018 (Table S2). *Eisenia fetida* accounted for 55% and 67% of the total density of earthworms in the deciduous and coniferous forests, respectively. The individuals of *E. fetida* added were excavated from nearby forests or bought from a farm in Jurong, China. Prior to adding to the mesocosms, they were kept in the respective forest soil for at least 30 days. Earthworms were picked by hand from the mesocosms and counted from May to August 2019 and the numbers were adjusted to the initial numbers added, i.e., 60 and 20 individuals in the deciduous and coniferous forest, respectively. Litterbags and soil samples were taken 70, 140, 210, 280, and 365 days after the installment of the mesocosms resulting in a total of 640 litterbags (2 forests × 4 deposited compounds treatments × 2 earthworm treatments × 2 mesh sizes × 4 replicates × 5 sampling dates) and 640 soil samples (0–5 cm depth underneath litterbags). At the 140‐day sampling, the nylon bag of one mesocosm in the deciduous forest was found broken and excluded from the analysis.

### 2.3 Litter mass loss, C, and N loss

The litter taken out of the litterbags was cleaned from debris using distilled water and dried at 60°C for 72 h. Total C and N of litter were measured from the samples taken after 70, 210, and 365 days using an elemental analyzer (Elemental Vario Micro). From these data changes in litter mass and amount of C and N were calculated and expressed as percentages of initial.

### 2.4 Soil pH and microbial biomass

Fresh soil samples were sieved through 1 mm and used to determine soil moisture and soil microbial biomass. Soil pH Was measured after adding 2.5 mL distilled water to 1 g dry‐weight soil, thoroughly mixing, and standing for 30 min using a pH meter (Mettler Toledo; Dick et al., 2000). Soil microbial biomass was determined by substrate‐induced respiration (SIR) following Bailey et al. (2002) and Lin et al. (2017). In brief, fresh soil samples equivalent to 1 g dry weight were adjusted to 60% of the water holding capacity. Then, 1 mL of glucose solution was added to achieve 10 mg glucose g^−1^ dry weight of soil. Samples were then incubated at 25°C, and CO_2_ was determined using an infrared gas analyzer after 1 h. SIR was expressed as CO_2_ per gram soil dry weight and hour.

### 2.5 Statistical analyses

All analyses were performed using R version 4.0.5 (https://www.r‐project.org/). We analyzed litter mass loss using linear mixed‐effects models (LMMs). Data from the deciduous and coniferous forests were analyzed separately. In each LMM, the type of deposited compounds (control, N, Na, PAHs), earthworms (with and without), mesh size (fine and coarse), and time (five sampling dates) were treated as fixed factors. Mesh size was nested in mesocosms and included as a random factor to account for the non‐independence of litterbags within mesocosms and repeated sampling. To evaluate the effects of the type of deposited compounds, earthworms, and mesh size, we used planned contrasts between the control and the respective treatment. Data were log(*x* + 1) transformed to meet normality if needed. Due to the response variables being log transformed, contrasts are analogous to log response ratios (Piovia‐Scott et al., 2019). We used ‘name’ to fit mixed‐effect models and ‘emmeans’ for planned contrast. The changes in litter mass loss, C, and N loss were shown as percentages and were calculated as $\left|m_{t}-m_{c}\right|\times 100\%$, with $m_{t}$ and $m_{c}$ the mass loss percentages of the treatment and control after 365 days, respectively. To evaluate the effects of the addition of *E. fetida*, we analyzed the abundance and biomass of earthworms at the end of the experiment using generalized linear models with quasi‐Poisson distribution using the ‘glm’ function to account for model over‐ or under‐dispersion.

Structural equation models (SEMs) were used to inspect pathways linking earthworms and deposited compounds to litter mass loss (see Figure 6 and Figure S4; Tian et al., 2018; Yin et al., 2022). To compare the effects of different types of deposited compounds and earthworms on litter mass loss, we merged six models for each forest as one including the three types of deposited compounds and two mesh sizes. In each model, deposited compounds and earthworms were included as categorical variables (with and without) and the other variables as numeric; values of all variables were scaled between 0–1 before modeling. Direct effects of Na on litter mass loss were included in the models of the coniferous forest according to the modification indices in R; direct effects of earthworms on SIR were removed to improve fitting (Shipley, 2009; Yin et al., 2022).

## 3 RESULTS

### 3.1 Changes in the abundance of earthworms

In both the deciduous and coniferous forests, the abundance, and biomass of total earthworms (including individuals potentially present before the addition of *E. fetida*) in the mesocosms with the earthworm addition was significantly higher than that in the control mesocosms by factors of 4.66 and 2.93 in deciduous and coniferous forests, respectively (*p* < .001, Table S1). Estimated survival rates of the added *E. fetida* were 12.1%, 11.7%, 7.1%, and 12.1% in the control, N, Na, and PAHs treatments in the deciduous forest and 17.5%, 17.5%, 28.8%, and 20.0% in the coniferous forest, respectively (Table S2).

### 3.2 Changes in litter mass loss, C, and N loss

**FIGURE 1:**
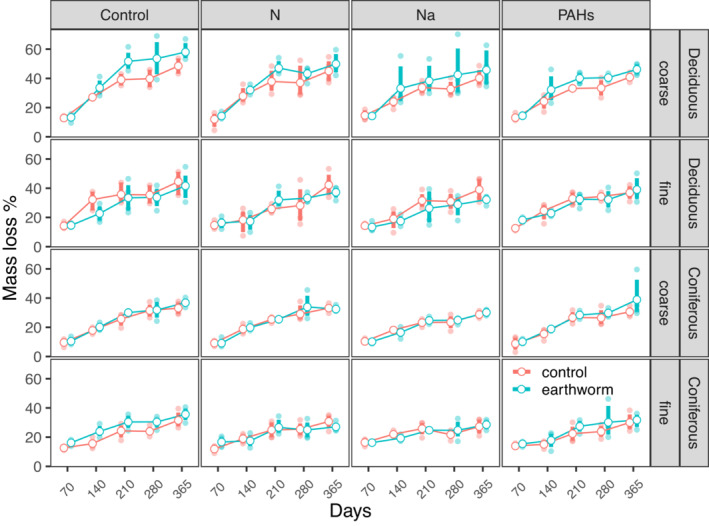
Changes in litter mass loss with time (70, 140, 210, 280, and 365 days) as affected by different types of deposited compounds (control, N, Na, and PAHs) and earthworms (with and without) in coarse and fine mesh size litterbags in deciduous and coniferous forests; means ± SE, *n* = 4. For the changes in litter total C and N loss see Figures S2 and S3, respectively.

**FIGURE 2:**
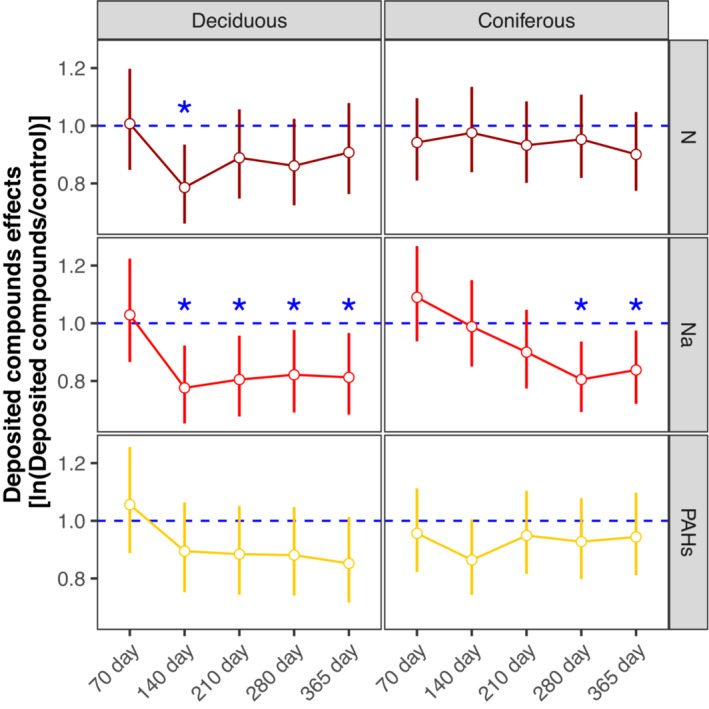
Changes in log response ratios [ln(deposited compounds/control)] of litter mass loss with time (70, 140, 210, 280, and 365 days) as affected by different types of deposited compounds (N, Na, and PAHs) in the deciduous (left) and coniferous forests (right); means with 95% confidence intervals; effect sizes were averaged across mesh size (coarse and fine) and earthworm treatment (with and without), *n* = 16; * indicate significant differences to the control (*p* < .05).

**FIGURE 3:**
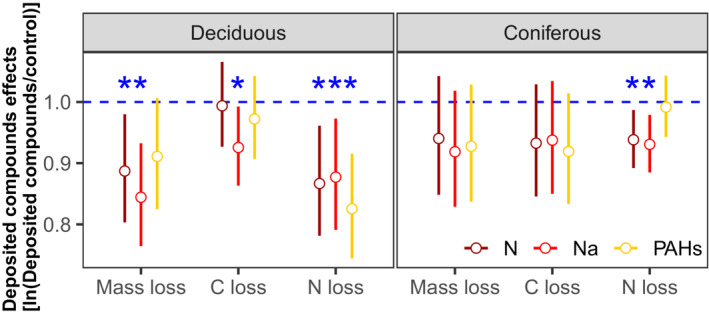
Effects of different types of deposited compounds (N, Na, and PAHs) on litter mass loss, C loss, and N loss in deciduous and coniferous forests; log response ratios [ln(deposited compounds/control)] equivalent to effect sizes ±95% confidence intervals; effect sizes were averaged across mesh size (coarse and fine), earthworm treatment (with and without) and sampling dates (70, 140, 210, 280, and 365 days), *n* = 80 for mass loss and *n* = 48 for C and N loss; * indicate significant differences to the control (*p* < .05).

Deposited compounds uniformly reduced litter mass loss in both deciduous and coniferous forests, with the effects being independent of mesh size (Figures 1 and 2; Table 2). The effects were strongest for Na, which decreased mass loss by 9.13% and 5.60% in the deciduous and coniferous forests, respectively (Table S3). Similar to mass loss, deposited compounds also reduced litter C and N loss (Table S4). Again, the effects of Na addition were strongest and reduced litter C loss by 7.15% in the deciduous forest and litter N loss by 7.63% in the coniferous forest (Figure 3; Table S3). The addition of N and PAHs reduced litter mass, C, and N loss but less than Na, e.g., N and PAHs reduced litter mass loss by 4.82%, 7.68% in the deciduous forest and by 3.42%, 1.46% in the coniferous forest, respectively (Figures 1, 2 and 3; Table S3).

**TABLE 2:** *F*‐ and *p*‐values of linear mixed effect models on the effects of deposited compounds (N, Na, and PAHs), earthworms (with and without), mesh size (fine and coarse), time (70, 140, 210, 280, and 365 days) and their interactions on litter mass loss in deciduous and conifer forests.

Factor	Deciduous			Coniferous		
	df	*F*	*p*	Df	*F*	*p*
(Intercept)	*1184*	40344.93	**<.001**	*1192*	30,229.79	**<.001**
Deposited compounds (D)	*3,23*	4.53	**.012**	*3,24*	1.17	.342
Earthworms (E)	*1,23*	5.28	**.031**	*1,24*	7.28	**.013**
Mesh size (M)	*1,23*	34.29	**<.001**	*1,24*	5.81	**.024**
Time (T)	*4184*	273.86	**<.001**	*4192*	341.1	**<.001**
D × E	*3,23*	0.89	.460	*3,24*	1.55	.227
D × M	*3,23*	1.37	.276	*3,24*	1.71	.192
E × M	*1,23*	9.41	**.005**	*1,24*	0.49	.492
D × T	*12,184*	1.09	.369	*12,192*	2.48	**.005**
E × T	*4184*	0.46	.766	*4192*	0.40	.806
M × T	*4184*	9.74	**<.001**	*4192*	29.83	**<.001**
D × E × M	*3,23*	0.64	.597	*3,24*	1.32	.292
D × E × T	*12,184*	0.62	.819	*12,192*	0.74	.712
D × M × T	*12,184*	1.17	0.306	*12,192*	0.62	.824
E × M × T	*4184*	1.32	0.262	*4192*	0.37	.829
D × E × M × T	*12,184*	0.47	0.929	*12,192*	0.90	.552

*Note:* The *p*‐values less than .05 were bolded.

**FIGURE 4:**
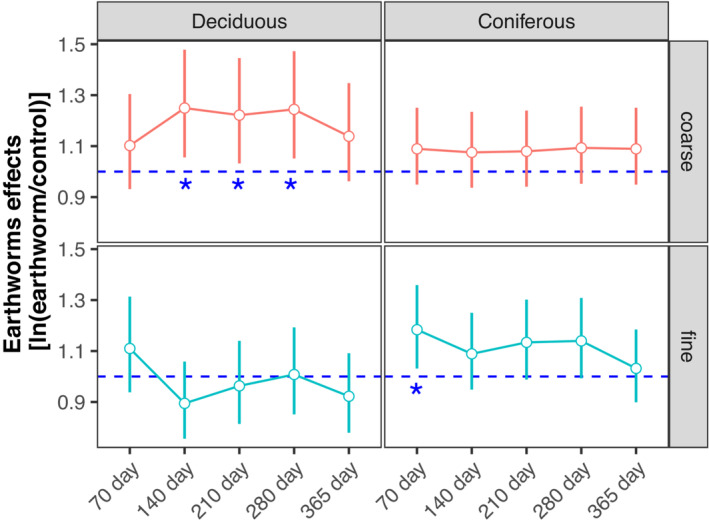
Changes in log response ratios [ln(earthworm/control)] of litter mass loss with time (70, 140, 210, 280, and 365 days) as affected by earthworms (with and without) and mesh (coarse and fine) in the deciduous (left) and conifer forests (right); means with 95% confidence intervals; effect sizes were averaged across deposited compounds treatments (control, N, Na, and PAHs), *n* = 16; * indicate significant differences to the control (*p* < .05).

**FIGURE 5:**
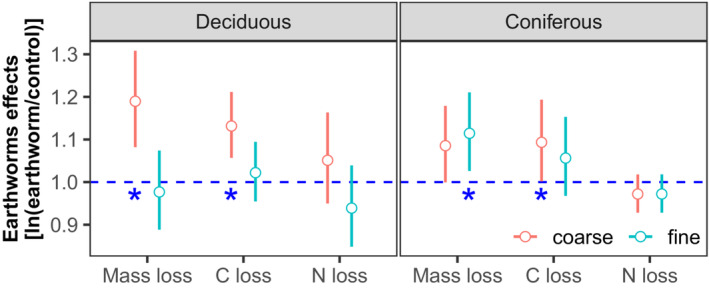
Changes in log response ratios [ln(earthworm/control)] of litter mass loss, C loss, and N loss as affected by mesh (meso‐ and macrofauna) and earthworms (with, without) in the deciduous (left) and coniferous forests (right); means with 95% confidence intervals; effect sizes were averaged across deposited compounds treatment (control, N, Na, and PAHs) and sampling dates (70, 140, 210, 280, and 365 days), *n* = 80 for mass loss and *n* = 48 for C and N loss; * indicate significant differences to the control (*p* < .05).

Earthworms generally accelerated litter mass loss in both deciduous and coniferous forests, with the effect being independent of deposited compounds (Figures 1 and 4; Table 2). The positive effect of earthworms on the loss of litter mass and litter C was similar but varied between forests. In the deciduous forest, earthworms significantly accelerated litter mass loss and C loss in coarse (6.66% and 6.20%) but not in fine mesh bags (Figures 4 and 5; Table S3). In the coniferous forest, earthworms significantly accelerated litter mass loss and C loss in both coarse and fine bags, but the effect was less strong than in the deciduous forest (2.02% and 2.80%, respectively; Figures 4 and 5; Tables S3 and S4).

### 3.3 Pathways affecting litter mass loss

**FIGURE 6:**
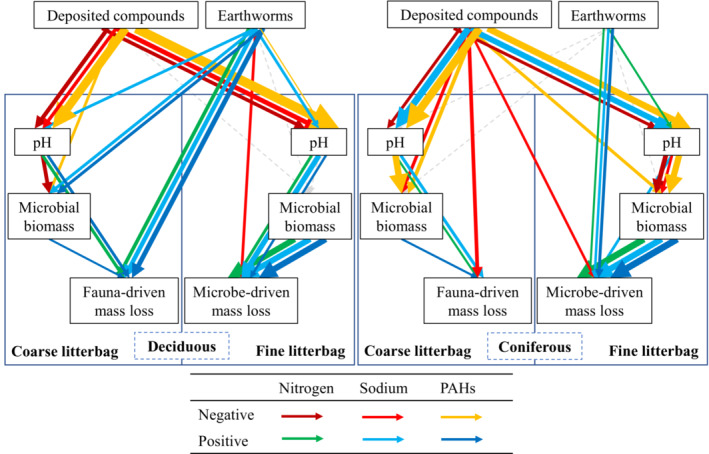
Structural Equation Models (SEMs) on the effect of earthworms and deposited compounds (N, Na, and PAHs; combined) on mass loss of litter in deciduous (left) and coniferous forests (right) via changes in soil pH and microbial biomass (for models on individual deposited compounds see Figure S4 and Table S4). Solid arrows represent marginally significant or significant relationships (*p* ≤ .1) and dashed gray arrows represent non‐significant relationships (*p* > .1). Dark red, red, and yellow arrows represent negative effects of N, Na, or PAHs, and green, light blue, and dark blue arrows represent positive effects. Arrow width is proportional to standardized path coefficients. Non‐standardized path coefficients associated with solid arrows are not shown (see Figure S4, Table S4); *n* = 80 (2 deposited compounds treatments × 2 earthworm treatments × 4 replicates × 5 sampling times); pH and microbial biomass refer to pH and microbial biomass in the soil underneath the litterbags. The fauna‐driven litter mass loss refers to the difference in litter mass loss between coarse and fine litterbags; microbial‐driven mass loss refers to the litter mass loss in fine litterbags. For the effects of deposited compounds on soil pH and microbial biomass see Figure S5.

The negative effects of deposited compounds on litter mass loss were mainly due to reduced soil pH in both the deciduous and coniferous forests (Figure 6; Figure S4; Table S5). Further, the addition of PAHs reduced animal‐driven litter mass loss by reducing soil microbial biomass in both the deciduous and coniferous forests. By contrast, in the coniferous forest, Na addition directly decreased litter mass loss driven by animals and microorganisms without changing soil pH and microbial biomass.

The pathways of positive earthworm effects on animal‐ and microbial‐driven mass loss varied with the types of deposited compounds and forests (Figure 6). Earthworms directly increased animal‐driven mass loss in the deciduous forest, but increased microbial‐driven mass loss in the coniferous forest. Further, in the deciduous forest, earthworms increased litter mass loss driven by animals and microorganisms by promoting soil pH in the Na addition treatment and by promoting soil microbial biomass in the PAH treatment.

## 4 DISCUSSION

Human activities increase the input of atmospheric compounds into natural ecosystems, with the compounds deposited including inorganic and non‐metal ions, metal ions, and organic contaminants (Li et al., 2016). These compounds are likely to differentially affect nutrient cycling in soil. Soil engineers, such as earthworms, play a key role in the nutrient cycling of terrestrial ecosystems (Blouin et al., 2013). However, studies investigating the interactive effect of deposited compounds and earthworms on nutrient cycling are lacking. This limits our understanding of the mechanism of deposited compounds affecting terrestrial C and N cycling and hampers the development of bioremediation strategies, e.g. by applying earthworms to contaminated forest soils. We investigated the modification of the effect of different types of deposited compounds on litter decomposition by earthworms. Unexpectedly, earthworms uniformly increased litter mass loss irrespective of the deposited compounds. Notably, the pathways earthworms increased litter mass loss varied among the different types of deposited compounds, likely reflecting that the decrease in litter mass loss by the different compounds was due to different mechanisms.

### 4.1 Effects of earthworms on litter mass loss irrespective of deposited compounds

As hypothesized, N, Na, and PAHs all decreased litter mass loss. Effects of deposited compounds on litter mass loss have been shown to vary with their concentrations, with detrimental effects typically increasing at higher concentrations (Ji et al., 2020; Knorr et al., 2005). Our N and PAHs treatments doubled the deposited amount of N and PAHs at our study sites, and the negative effects were in line with previous studies, but their effects were relatively small compared to the addition of Na. Fast cycling and losses of N via leaching (Kreutzer et al., 2009; Wang et al., 2021) might have been responsible for the weak N effect. Although increased by a factor of two, the concentration of PAHs in this study (1.813 μg g^−1^ dry soil) may have little toxic effects on soil decomposers according to previous studies (Rodriguez‐Campos et al., 2014; Zhang, Chao, et al., 2016). Further, earthworms and lignin‐degrading fungi, known to stimulate the degradation of PAHs (Haritash & Kaushik, 2009; Rodriguez‐Campos et al., 2014), may have contributed to the weak effects of PAHs on litter mass loss. Compared to N and PAHs, Na addition more strongly reduced litter mass loss, potentially due to the rather high concentrations of Na added (0.5% NaCl solution, 3.28 g Na^+^ m^−2^ month^−1^; Jia et al., 2015; Kaspari et al., 2009, 2014). In the region of Nanjing, the input of Na of predominantly natural (marine) origin into terrestrial ecosystems is only 40.8 μg m^−2^ month^−1^ (Li et al., 2016). The results support earlier findings that in contrast to small Na input, high amounts of Na inhibit both faunal and microbial activity, and thereby decrease litter mass loss (Jia et al., 2015). Overall, the results suggest that the effect of deposited compounds on litter mass loss depends on the type of compounds with the effects of low concentrations of deposited compounds affecting litter mass loss in subtropical forest ecosystems only moderately.

Notably, the positive effect of earthworms on litter mass loss in both the deciduous and coniferous forests was not significantly modified by deposited compounds, which contradicts our second hypothesis. Presumably, the low sensitivity of *E. fetida* to soil contaminants contributed to the consistent effect on litter mass loss. In fact, *E. fetida* is known to be able to live in highly contaminated soils (Geissen et al., 2008) suggesting that it is rather insensitive to soil contaminations and may even contribute to the decontamination of soils (Rodriguez‐Campos et al., 2014). Effects of earthworms on litter mass loss have been shown to vary among ecological groups of earthworms, with the effects of epigeic species such as *E. fetida* being strong and exceeding those of other ecological groups of earthworms (Heungens, 1969; Rajapaksha et al., 2013; Suarez et al., 2006). Our results indicate that these effects are rather insensitive to deposited compounds, although at high concentrations of PAHs and Na, they are likely to be detrimentally affected (Jia et al., 2015; Peng et al., 2008).

### 4.2 Pathways linking the effect of deposited compounds and earthworms to litter mass loss

Partly supporting our third hypothesis, our SEMs indicated that N, Na, and PAHs all indirectly inhibited litter mass loss in the deciduous forest by acidifying the soil. By contrast, they little affected soil microbial biomass in particular in fine litterbags suggesting that deposited compounds changed the activity rather than the biomass of microorganisms. N and PAHs also indirectly inhibited animal‐driven litter mass loss by acidifying the soil. Considering that N addition has been found to little affect animal‐driven mass loss (Zhang et al., 2016), the effects were likely indirect via soil acidification (Lin et al., 2017), potentially due to deteriorating environmental requirements of detritivores. Notably, PAHs also indirectly inhibited animal‐driven litter mass loss by reducing microbial biomass in both forests. The lack of effects of Na on animal‐driven litter mass loss may have been due to the detrimental effects of high concentrations of Na being canceled out by Na functioning as an essential element for soil animals (Kaspari et al., 2014). Results of our SEMs further indicated that in the coniferous forest, Na directly inhibited litter mass loss suggesting that it also affects animal and microbial activity. Overall, our SEMs suggest that deposited compounds acidified the soil and thereby inhibited the activity of soil detritivores and microorganisms, but the pathways of the detrimental effects of these compounds on litter mass loss varied among the types of deposited compounds. In the long‐term detrimental effects of PAHs are likely to exceed the negative effects of N and Na due to their accumulation in litter and decomposer organisms (Jonker & van der Heijden, 2007; Muijs & Jonker, 2009).

Earthworms increased litter mass loss by directly affecting faunal‐ and microbial‐driven litter mass loss, and indirectly affecting soil pH and soil microbial biomass, supporting our third hypothesis. However, the pathways to how earthworms promoted litter mass loss differed between forests and corresponded to the different responses of earthworms to deposited compounds. Earthworms directly promoted animal‐driven litter mass loss in the deciduous forest, but promoted microbial‐driven litter mass loss in the coniferous forest. Since earthworms prefer to feed on high‐quality litter (Rajapaksha et al., 2013), and earthworm abundance and litter mass loss correlate positively (Huang et al., 2020), earthworm abundance and litter quality may have contributed to the different effects of earthworms on litter mass loss in the deciduous and coniferous forest. Further, the mitigation of the negative effect of Na on litter mass loss in the deciduous forest by earthworms likely was due to earthworms reducing the negative effect of Na on soil pH. Earlier studies also found earthworms to alter soil pH (Sackett et al., 2013), but our results indicate that this may depend on the type of deposited compounds. By contrast, according to our SEM, the mitigation of the negative effect of the addition of N on litter mass loss (and soil pH) in the coniferous forest by earthworms was due to earthworms directly increasing litter mass loss. Further, the negative effect of PAHs on animal‐driven litter mass loss was mitigated by earthworms via beneficially affecting pH and microbial biomass, but also directly by increasing litter mass loss.

Although earthworms uniformly stimulated litter mass loss in both the deciduous and coniferous forests, the pathways they stimulated litter mass loss differed between the two forests. These differences are likely to be related to different soil C and N contents in the two forests. As we did not replicate forest types and only studied a single deciduous (*Q. variabilis*) and a single coniferous (*P. massoniana*) forest, the differences cannot be ascribed to differences in forest type. Also hampering the comparison of the different forest types, we added a larger number of earthworms to the deciduous than to the coniferous forest to study the effects of the actual earthworm density in respective forests. It has been shown previously that the effect of earthworms on litter mass loss increases with earthworm abundance (Gonzalez et al., 2003; Huang et al., 2020; Szlavecz et al., 2011).

## 5 CONCLUSION

This study provided novel and detailed insight into how atmospheric‐deposited compounds and earthworms affect nutrient cycling in interactive ways. Importantly, using SEMs we identified mechanisms and pathways responsible for these interactions. Our results suggest that the positive effects of earthworms on litter mass loss are not modified by different types of deposited compounds, but the mechanisms they affect litter mass loss vary with the types of deposited compounds. Although not replicated, the results further suggest that with the addition of different types of deposited compounds, the mechanisms responsible for the uniform and positive effects of earthworms on litter mass loss vary with forest type. The results highlight the importance of studying the effects of deposited compounds and earthworms on terrestrial element cycling in concert as the beneficial roles of earthworms may persist and uniformly mitigate the detrimental effects of deposited compounds.

## AUTHOR CONTRIBUTIONS

**Junbo Yang:** Conceptualization (equal); data curation (equal); formal analysis (equal); investigation (equal); methodology (equal); visualization (equal); writing – original draft (equal); writing – review and editing (equal). **Kai Tian:** Conceptualization (equal); investigation (equal); methodology (equal); software (equal); writing – review and editing (equal). **Jingzhong Lu:** Conceptualization (equal); methodology (equal); software (equal); visualization (equal); writing – review and editing (equal). **Xiangshi Kong:** Investigation (equal); validation (equal); writing – review and editing (equal). **Qiang Li:** Investigation (equal); validation (equal); writing – review and editing (equal). **Rumeng Ye:** Investigation (equal); validation (equal). **Xiaoyi Zeng:** Investigation (equal); validation (equal); writing – review and editing (equal). **Tingting Cao:** Investigation (equal); validation (equal); writing – review and editing (equal). **Haijing Hu:** Investigation (equal); validation (equal). **Yanli Ji:** Investigation (equal); validation (equal). **Xingjun Tian:** Conceptualization (equal); data curation (equal); funding acquisition (equal); project administration (equal); resources (equal); supervision (equal); writing – review and editing (equal). **Stefan Scheu:** Conceptualization (equal); writing – review and editing (equal).

## CONFLICT OF INTEREST STATEMENT

The authors declare that they have no conflict of interest.

## Supporting information


Figure S1

Figure S2

Figure S3

Figure S4

Figure S5

Tables S1‐S5

Data S1

## Data Availability

The data that support the findings of this study are openly available in the Dryad Digital Repository at https://doi.org/10.5061/dryad.b5mkkwhhc.
