# Supplementary material for: Earthworms increase forest litter mass loss irrespective of deposited compounds – A field manipulation experiment in subtropical forests
Source: Ecol Evol. 2023 Apr 30;13(5):e10047. doi: 10.1002/ece3.10047 (PMC10150166; doi:10.1002/ece3.10047)
Supplement: Supplementary file 1 — Figure S1 [file ECE3-13-e10047-s002.pdf]

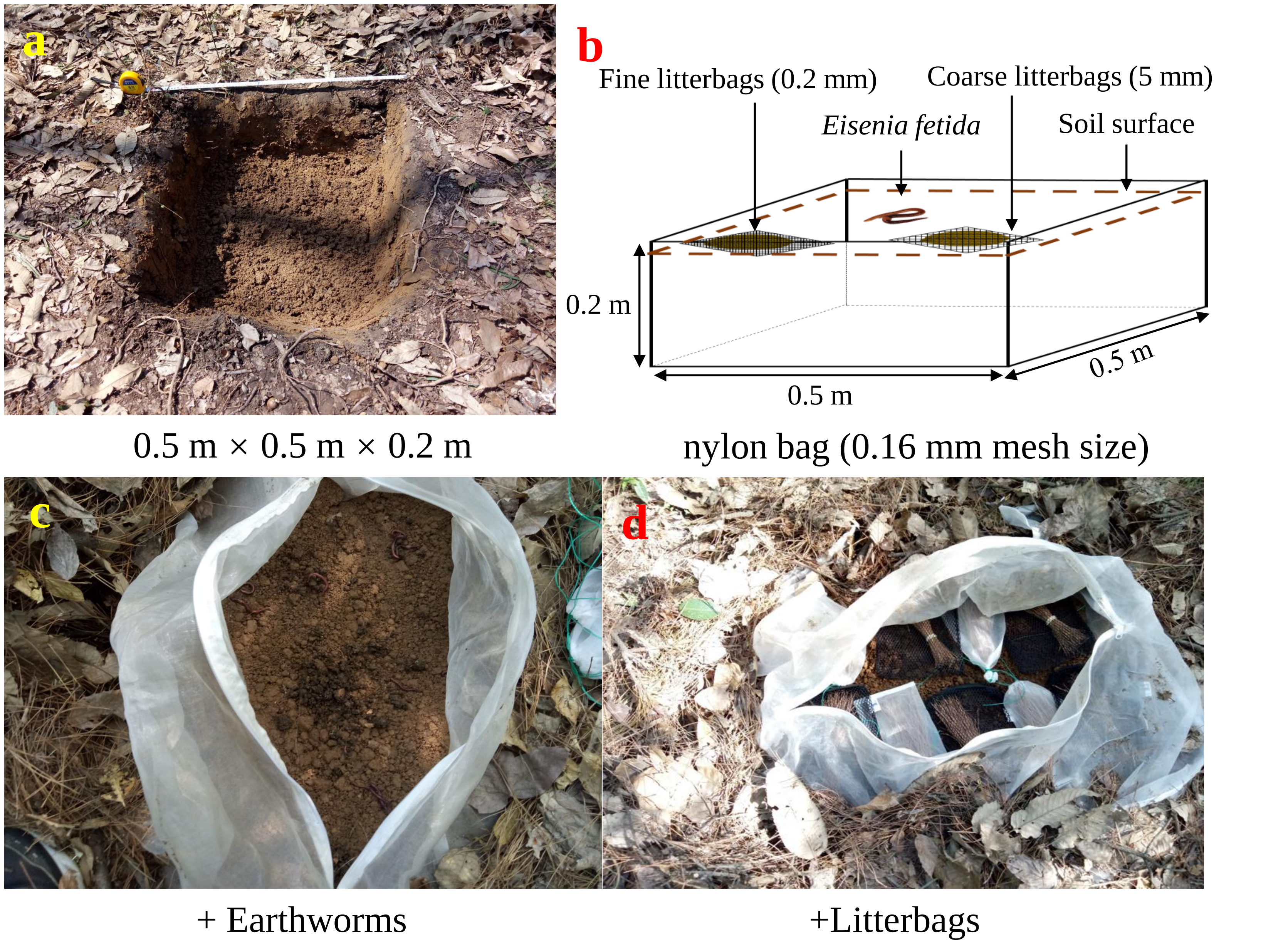

Fig. S1 Set-up of the mesocosms; (a) excavation of soil pit of 0.5 m × 0.5 m × 0.2 m; (b) the diagram of nylon bag (mesocosms) with earthworms, coarse and fine litter bags; (c) addition of *Eisenia fetida* individuals to the mesocosms; (d) placement of litterbags into the mesocosms.
