# Supplementary material for: Earthworms increase forest litter mass loss irrespective of deposited compounds – A field manipulation experiment in subtropical forests
Source: Ecol Evol. 2023 Apr 30;13(5):e10047. doi: 10.1002/ece3.10047 (PMC10150166; doi:10.1002/ece3.10047)
Supplement: Supplementary file 2 — Figure S2 [file ECE3-13-e10047-s004.pdf]

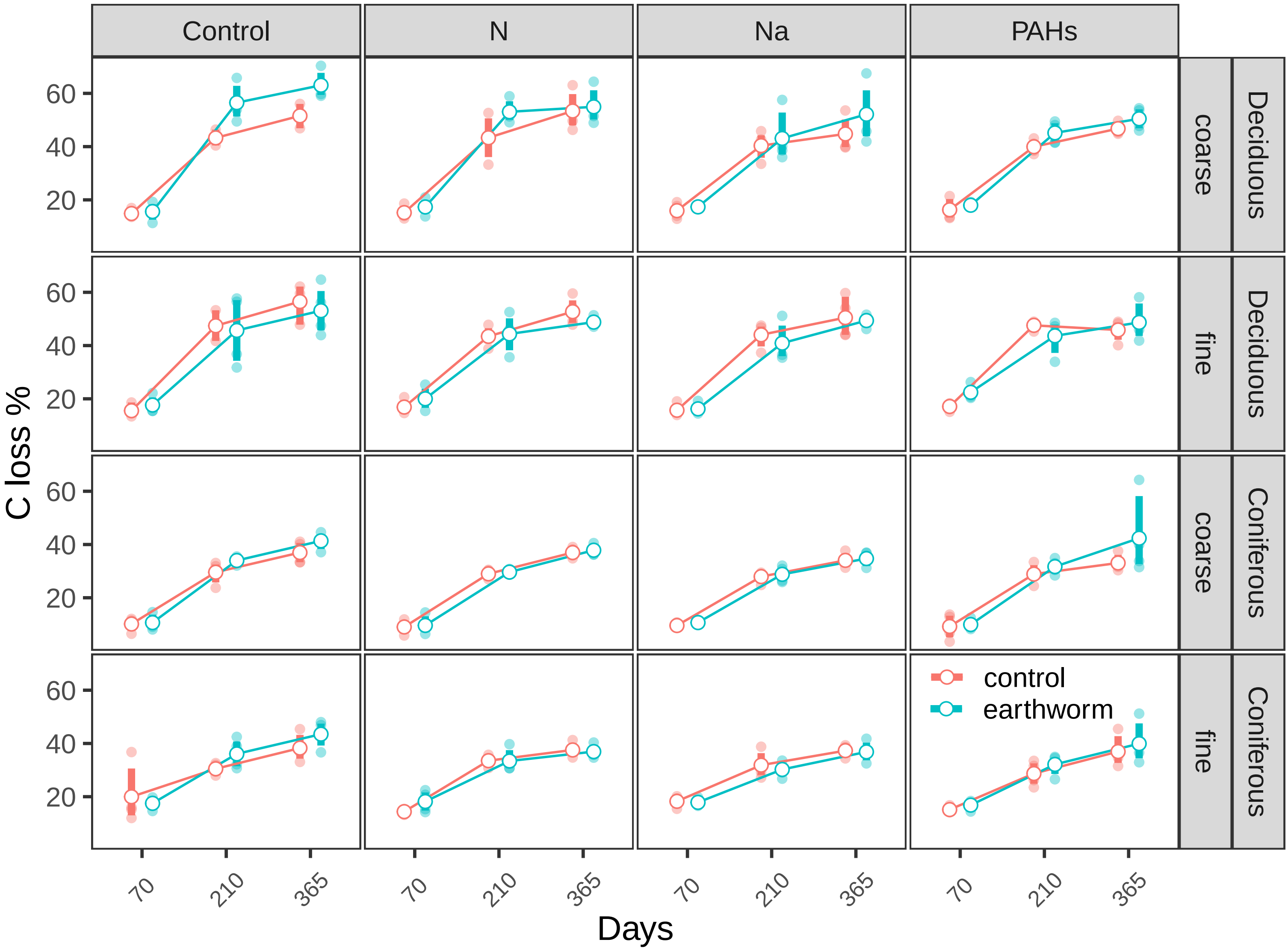

Fig. S2 Litter total C loss as affected by type of deposited compounds (Control, N, Na, PAHs) and addition of earthworms (with, green; without, red) in litterbags with coarse and fine mesh size in deciduous and coniferous forests; means ± SE; n = 4.
