# Supplementary figures and images for: Earthworms increase forest litter mass loss irrespective of deposited compounds – A field manipulation experiment in subtropical forests
Source: Ecol Evol. 2023 Apr 30;13(5):e10047. doi: 10.1002/ece3.10047 (PMC10150166; doi:10.1002/ece3.10047)

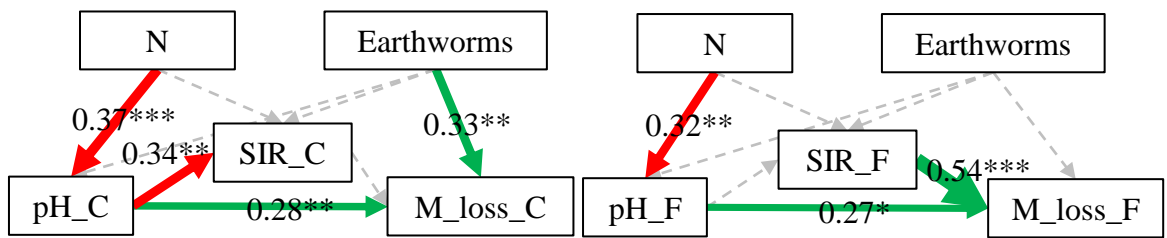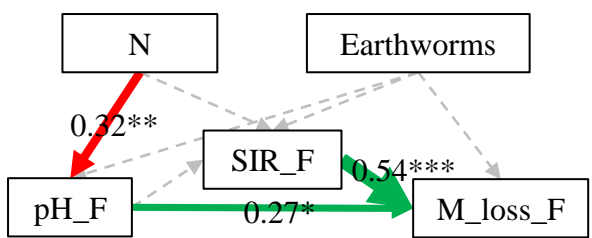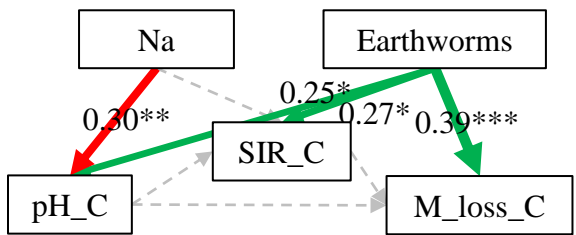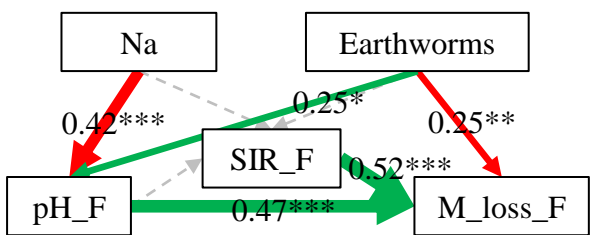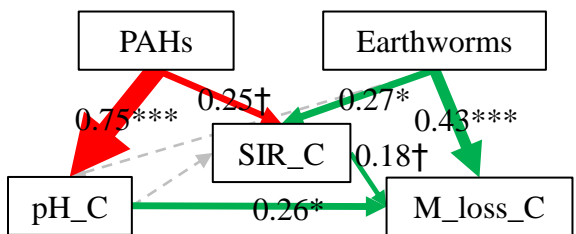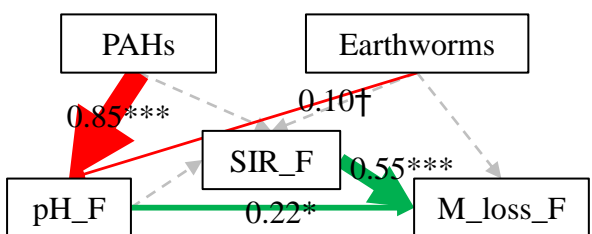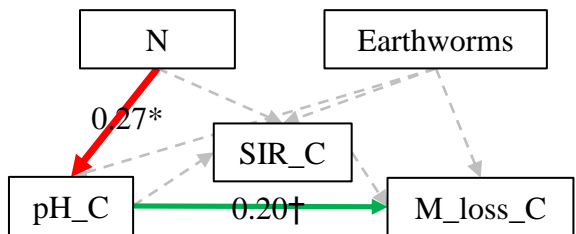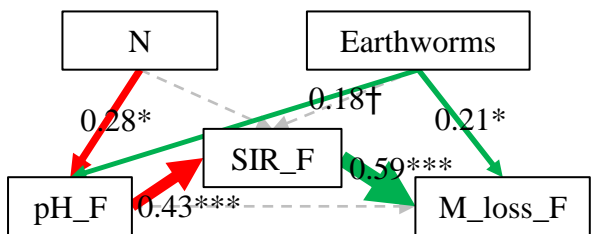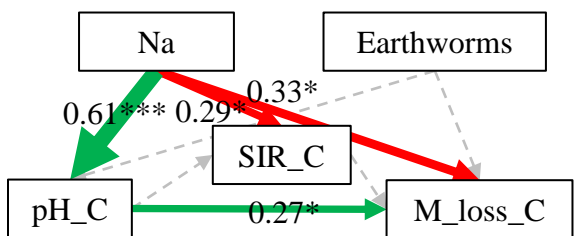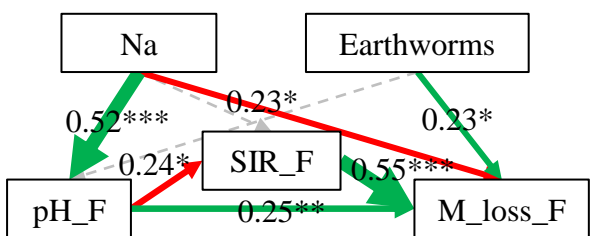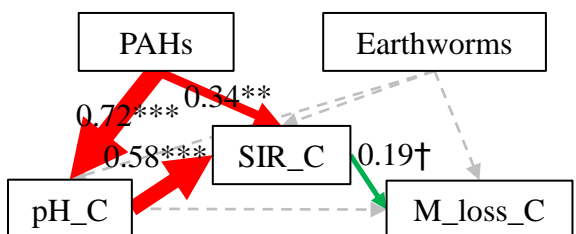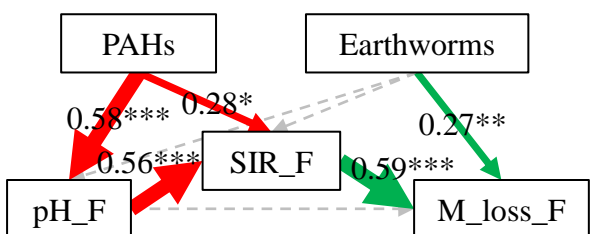

Supplement: Supplementary file 4 — Figure S4 [file ECE3-13-e10047-s006.pdf]
