# Supplementary material for: Earthworms increase forest litter mass loss irrespective of deposited compounds – A field manipulation experiment in subtropical forests
Source: Ecol Evol. 2023 Apr 30;13(5):e10047. doi: 10.1002/ece3.10047 (PMC10150166; doi:10.1002/ece3.10047)
Supplement: Supplementary file 5 — Figure S5 [file ECE3-13-e10047-s007.pdf]

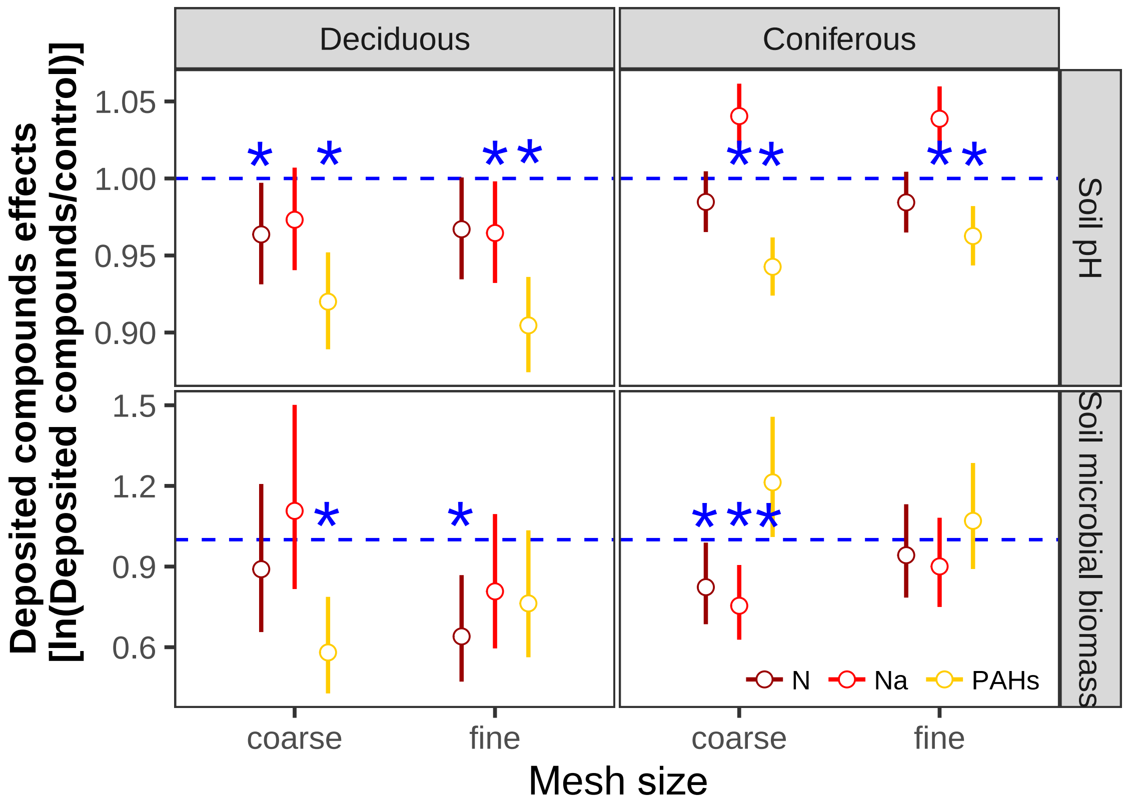

Figure S5 Effects of different types of deposited compounds (N, Na, PAHs) and mesh sizes (coarse and fine) on soil pH and microbial biomass in deciduous and coniferous forests; log response ratios [ln(deposited compounds/control)] equivalent to effect sizes ± 95% confidence intervals; effect sizes were averaged across earthworm treatment (with and without) and sampling dates (70, 140, 210, 280, 365 days), n = 40 for mass loss and n = 24 for C and N loss; asterisks indicate significant differences to the control ($P < 0.05$).
