# Supplementary material for: Earthworms increase forest litter mass loss irrespective of deposited compounds – A field manipulation experiment in subtropical forests
Source: Ecol Evol. 2023 Apr 30;13(5):e10047. doi: 10.1002/ece3.10047 (PMC10150166; doi:10.1002/ece3.10047)
Supplement: Supplementary file 6 — Tables S1‐S5 [file ECE3-13-e10047-s003.docx]

**Appendix**

Tables

**Table S1**

The difference in the abundance and biomass of total earthworms and of *Eisenia fetida* among deposited compound (D) and earthworm (E) treatments in mesocosms of deciduous and coniferous forests; Chi^2^- and P-values based on generalized linear models.

|  |  | **Deciduous** | |  | **Coniferous** | |
| --- | --- | --- | --- | --- | --- | --- |
|  | Df | Chi^2^ | P |  | Chi^2^ | P |
| **Total abundance** |  |  |  |  |  |  |
| Deposited compounds (D) | 3 | 3.18 | 0.364 |  | 11.96 | **0.008** |
| Earthworms (E) | 1 | 19.83 | **<0.001** |  | 14.14 | **<0.001** |
| D × E | 3 | 0.86 | 0.835 |  | 1.43 | 0.698 |
| **Total biomass** | |  |  |  |  |  |
| Deposited compounds (D) | 3 | 5.59 | 0.134 |  | 20.27 | **<0.001** |
| Earthworms (E) | 1 | 27.04 | **<0.001** |  | 19.99 | **<0.001** |
| D × E | 3 | 0.14 | 0.987 |  | 2.42 | 0.490 |
| ***Eisenia* abundance** | |  |  |  |  |  |
| Deposited compounds (D) | 3 | 1.11 | 0.775 |  | 4.26 | 0.234 |
| Earthworms (E) | 1 | 18.02 | **<0.001** |  | 17.81 | **<0.001** |
| D × E | 3 | 1.65 | 0.648 |  | 5.44 | 0.142 |
| ***Eisenia* biomass** | |  |  |  |  |  |
| Deposited compounds (D) | 3 | 2.98 | 0.394 |  | 4.14 | 0.246 |
| Earthworms (E) | 1 | 17.67 | **<0.001** |  | 15.59 | **<0.001** |
| D × E | 3 | 0.69 | 0.875 |  | 4.07 | 0.254 |

**Table S2**

Total abundance and biomass of earthworms in mesocosms of the deciduous (D) and coniferous (C) forest with and without addition of *E. fetida* (E); means ± SD, n = 5 for initial values and n = 4 for other values.

|  | **Initial** | **Control** | **N addition** | **Na addition** | **PAHs addition** |
| --- | --- | --- | --- | --- | --- |
| **Total abundance** (number per mesocosm) | | | | | |
| D | 60.60 ± 9.71 | 3.67 ± 6.35 | 3.75 ± 4.86 | 1.25 ± 1.26 | 2.00 ± 2.16 |
| D+E |  | 10.00 ± 4.69 | 15.75 ± 8.73 | 8.00 ± 4.24 | 14.75 ± 14.08 |
| C | 19.60 ± 8.08 | 0.50 ± 0.58 | 1.75 ± 2.36 | 4.00 ± 4.62 | 1.25 ± 1.50 |
| C+E |  | 3.75 ± 3.20 | 4.00 ± 0.82 | 9.50 ± 5.00 | 4.75 ± 1.26 |
| ***Eisenia* abundance** (number per mesocosm) | | | | | |
| D | 31.00 ± 7.18 | 0 | 1.50 ± 1.73 | 0.25 ± 0.50 | 0.25 ± 0.50 |
| D+E |  | 7.25 ± 2.75 | 7.00 ± 10.80 | 4.25 ± 3.40 | 7.25 ± 9.98 |
| C | 12.20 ± 5.07 | 0 | 1.75 ± 2.36 | 2.00 ± 2.45 | 1.00 ± 1.41 |
| C+E |  | 3.50 ± 2.89 | 3.50 ± 1.29 | 5.75 ± 2.63 | 4.00 ± 1.41 |
| **Total biomass** (g per mesocosm) | | | | | |
| D | 1.69 ± 0.41 | 0.04 ± 0.06 | 0.07 ± 0.05 | 0.03 ± 0.03 | 0.04 ± 0.06 |
| D+E |  | 0.50 ± 0.37 | 0.95 ± 1.02 | 0.30 ± 0.08 | 0.97 ± 1.13 |
| C | 0.81 ± 0.25 | 0.01 ± 0.01 | 0.05 ± 0.06 | 0.06 ± 0.07 | 0.04 ± 0.07 |
| C+E |  | 0.08 ± 0.05 | 0.11 ± 0.03 | 0.41 ± 0.28 | 0.12 ± 0.07 |
| ***Eisenia* biomass** (g per mesocosm) | | | | | |
| D | 1.18 ± 0.28 | 0 | 0.05 ± 0.05 | 0.01 ± 0.02 | 0.01 ± 0.01 |
| D+E |  | 0.36 ± 0.17 | 0.52 ± 0.95 | 0.22 ± 0.13 | 0.72 ± 0.99 |
| C | 0.59 ± 0.27 | 0 | 0.05 ± 0.06 | 0.04 ± 0.05 | 0.04 ± 0.07 |
| C+E |  | 0.08 ± 0.05 | 0.09 ± 0.05 | 0.14 ± 0.07 | 0.11 ± 0.07 |

**Table S3**

Litter mass loss, total C and N loss as affected by the addition of earthworms (without, control; with, earthworm), mesh size (coarse, fine) and the type of deposited compounds (control, N, Na, PAHs) at 365 days in deciduous and coniferous forests; mean ± SD.

| **Forests** | **Earthworm** | **Mesh, deposition** | **n** | **Mass loss %** | **C loss %** | **N loss %** |
| --- | --- | --- | --- | --- | --- | --- |
| **Deciduous** | control |  | 30 | 41.85 ± 6.89 | 50.00 ± 6.44 | 33.08 ± 6.89 |
|  | earthworm |  | 32 | 43.73 ± 10.16 | 52.57 ± 7.45 | 34.70 ± 9.74 |
|  | control | coarse | 15 | 43.28 ± 6.27 | 48.95 ± 6.16 | 34.22 ± 6.77 |
|  |  | fine | 15 | 40.42 ± 7.40 | 51.06 ± 6.76 | 31.95 ± 7.07 |
|  | earthworm | coarse | 16 | 49.94 ± 8.96 | 55.14 ± 8.27 | 40.16 ± 9.46 |
|  |  | fine | 16 | 37.52 ± 7.14 | 50.00 ± 5.67 | 29.25 ± 6.56 |
|  | all | control | 14 | 48.40 ± 9.62 | 56.34 ± 7.80 | 39.46 ± 9.63 |
|  |  | N | 16 | 43.58 ± 8.06 | 52.46 ± 5.61 | 33.64 ± 7.31 |
|  |  | Na | 16 | 39.27 ± 8.95 | 49.19 ± 7.42 | 32.39 ± 9.48 |
|  |  | PAHs | 16 | 40.72 ± 5.99 | 47.94 ± 4.54 | 30.89 ± 5.11 |
|  |  |  |  |  |  |  |
| **Coniferous** | control |  | 32 | 30.65 ± 4.52 | 36.39 ± 3.74 | 17.76 ± 6.79 |
|  | earthworm |  | 32 | 32.67 ± 6.51 | 39.19 ± 6.57 | 15.71 ± 10.32 |
|  | control | coarse | 16 | 31.42 ± 3.55 | 35.26 ± 3.31 | 21.28 ± 3.92 |
|  |  | fine | 16 | 29.87 ± 5.32 | 37.52 ± 3.90 | 14.25 ± 7.32 |
|  | earthworm | coarse | 16 | 34.60 ± 7.41 | 39.08 ± 7.69 | 20.14 ± 8.02 |
|  |  | fine | 16 | 30.74 ± 4.97 | 39.30 ± 5.47 | 11.27 ± 10.68 |
|  | all | control | 16 | 34.27 ± 4.61 | 40.02 ± 4.81 | 20.26 ± 4.58 |
|  |  | N | 16 | 30.86 ± 3.95 | 37.33 ± 2.11 | 14.28 ± 7.40 |
|  |  | Na | 16 | 28.68 ± 3.40 | 35.73 ± 2.97 | 12.64 ± 10.93 |
|  |  | PAHs | 16 | 32.82 ± 8.17 | 38.08 ± 8.93 | 19.77 ± 8.81 |

**Table S4**

F- and P-values of linear mixed-effect models on the effects of deposited compounds (N, Na, PAHs), earthworms (with, without), mesh size (small, large) and time (70, 140, 210, 280, 365 days) on changes in litter C and N in deciduous and coniferous forests.

| **Factor** | **Deciduous** | | | | | **Coniferous** | | | | |
| --- | --- | --- | --- | --- | --- | --- | --- | --- | --- | --- |
|  |  | C loss | | N loss | |  | C loss | | N loss | |
|  | df | F | P | F | P | df | F | P | F | P |
| (Intercept) | *1,92* | 91514.6 | **<0.001** | 962.5 | **<0.001** | *1,96* | 36201.2 | **<0.001** | 265.5 | **<0.001** |
| Deposited compounds (D) | *3,23* | 2.48 | 0.086 | 3.41 | **0.034** | *3,24* | 1.23 | 0.320 | 3.29 | **0.038** |
| Earthworms (E) | *1,23* | 9.65 | **0.005** | 0.00 | 0.945 | *1,24* | 4.60 | **0.042** | 3.19 | 0.087 |
| Mesh size (M) | *1,23* | 0.17 | 0.685 | 28.35 | **<0.001** | *1,24* | 75.82 | **<0.001** | 16.19 | **<0.001** |
| Time (T) | *2,92* | 1274.58 | **<0.001** | 75.33 | **<0.001** | *2,96* | 744.49 | **<0.001** | 0.52 | 0.594 |
| D x E | *3,23* | 0.25 | 0.863 | 4.27 | **0.015** | *3,24* | 0.61 | 0.615 | 0.62 | 0.606 |
| D x M | *3,23* | 0.62 | 0.610 | 0.34 | 0.794 | *3,24* | 0.13 | 0.942 | 2.40 | 0.093 |
| E x M | *1,23* | 4.54 | **0.044** | 0.12 | 0.728 | *1,24* | 0.44 | 0.512 | 0.87 | 0.361 |
| D x T | *6,92* | 3.48 | **0.004** | 0.67 | 0.675 | *6,96* | 1.15 | 0.340 | 1.67 | 0.137 |
| E x T | *2,92* | 1.69 | 0.191 | 0.03 | 0.967 | *2,96* | 0.06 | 0.939 | 0.11 | 0.895 |
| M x T | *2,92* | 3.11 | **0.049** | 0.82 | 0.442 | *2,96* | 51.36 | **<0.001** | 0.65 | 0.526 |
| D x E x M | *3,23* | 0.38 | 0.766 | 2.29 | 0.106 | *3,24* | 0.24 | 0.865 | 0.65 | 0.588 |
| D x E x T | *6,92* | 0.85 | 0.535 | 3.49 | **0.004** | *6,96* | 0.57 | 0.750 | 1.20 | 0.311 |
| D x M x T | *6,92* | 1.32 | 0.256 | 1.17 | 0.330 | *6,96* | 0.23 | 0.967 | 3.00 | **0.010** |
| E x M x T | *2,92* | 4.03 | **0.021** | 3.60 | **0.031** | *2,96* | 0.04 | 0.964 | 0.67 | 0.514 |
| D x E x M x T | *6,92* | 0.41 | 0.869 | 1.47 | 0.198 | *6,96* | 0.29 | 0.940 | 0.97 | 0.450 |

**Table S5**

Fit values of structural equation modeling on the effect of deposited compounds (N, nitrogen; Na, sodium; PAHs, polycyclic aromatic hydrocarbons) and earthworms (micro, without; fauna, with) in deciduous (D) and coniferous forest (C). P indicates the p-value of models, X^2^/df indicates the ratio of chi-square and degrees of freedom, GFI indicates the goodness of fit, RMSEA indicates the root mean square error of approximation, P__RMSEA_ indicates the p-value of RMSEA, R^2^_Loss, R^2^_SIR and R^2^_pH indicates the estimate of variances of mass loss, soil microbial biomass and soil pH, respectively.

|  | P | X^2^/df | GFI | RMSEA | P__RMSEA_ | R^2^_Loss | R^2^_SIR | R^2^_pH |
| --- | --- | --- | --- | --- | --- | --- | --- | --- |
| D_N_fauna | 0.112 | 2.530 | 0.979 | 0.143 | 0.145 | 0.036 | 0.025 | 0.036 |
| D_N_micro | 0.834 | 0.044 | 1.000 | 0.000 | 0.849 | 0.036 | 0.071 | 0.043 |
| C_N_fauna | 0.151 | 2.060 | 0.983 | 0.115 | 0.191 | 0.040 | 0.081 | 0.026 |
| C_N_micro | 0.368 | 0.810 | 0.993 | 0.000 | 0.414 | 0.038 | 0.057 | 0.037 |
| D_Na_fauna | 0.939 | 0.006 | 1.000 | 0.000 | 0.945 | 0.028 | 0.037 | 0.032 |
| D_Na_micro | 0.926 | 0.009 | 1.000 | 0.000 | 0.933 | 0.029 | 0.064 | 0.033 |
| C_Na_fauna | 0.613 | 0.256 | 0.998 | 0.000 | 0.647 | 0.053 | 0.075 | 0.025 |
| C_Na_micro | 0.854 | 0.034 | 1.000 | 0.000 | 0.868 | 0.034 | 0.072 | 0.029 |
| D_PAHs_fauna | 0.803 | 0.062 | 1.000 | 0.000 | 0.820 | 0.033 | 0.039 | 0.026 |
| D_PAHs_micro | 0.115 | 2.479 | 0.994 | 0.140 | 0.149 | 0.039 | 0.056 | 0.018 |
| C_PAHs_fauna | 0.882 | 0.022 | 1.000 | 0.000 | 0.893 | 0.020 | 0.039 | 0.026 |
| C_PAHs_micro | 0.170 | 1.880 | 0.986 | 0.105 | 0.212 | 0.031 | 0.043 | 0.026 |
