# Supplementary material for: Earthworms increase forest litter mass loss irrespective of deposited compounds – A field manipulation experiment in subtropical forests
Source: Ecol Evol. 2023 Apr 30;13(5):e10047. doi: 10.1002/ece3.10047 (PMC10150166; doi:10.1002/ece3.10047)
Supplement: Supplementary file 7 — Data S1 [file ECE3-13-e10047-s001.pdf]

Fig. S4 Structural equation models on the effects of earthworm and types of deposited compounds (N, Na, PAHs) on fauna-driven (large mesh size litterbags) and microbial-driven decomposition (small mesh size litterbags) in deciduous (upper six graphs) and coniferous forest (lower six graphs). Arrows represent causal pathways. Solid arrows represent marginally significant relationships ($P \leq 0.1$), dashed grey arrows represent non-significant relationships ($P > 0.1$). Red arrows refer to negative effects, green arrows to positive effects. Arrow width represents the standardized path coefficients. Non-standardized path coefficients associated with each solid arrow are shown (†)$P \leq 0.1$, (*)$P \leq 0.05$, (**)$P \leq 0.01$, (***)$P \leq 0.001$; $n = 80$ (2 deposited compounds treatments × 2 earthworm treatments × 4 replicates × 5 sampling times). SIR_C, microbial biomass in soil underneath coarse litterbags, pH_C, soil pH underneath coarse litterbags, SIR_F, microbial biomass in soil underneath fine litterbags, pH_F, soil pH underneath fine litterbags, M_loss_C, fauna-driven litter decomposition (i.e., the difference in mass loss between coarse and fine litterbags), M_loss_F, microbe-driven litter mass loss (fine litterbags).
